# How do core autism traits and associated symptoms relate to quality of life? Findings from the Longitudinal European Autism Project

**DOI:** 10.1177/1362361320959959

**Published:** 2020-10-07

**Authors:** Bethany FM Oakley, Julian Tillmann, Jumana Ahmad, Daisy Crawley, Antonia San José Cáceres, Rosemary Holt, Tony Charman, Tobias Banaschewski, Jan Buitelaar, Emily Simonoff, Declan Murphy, Eva Loth

**Affiliations:** 1 King’s College London, UK; 2 University of Vienna, Austria; 3 University of Greenwich, UK; 4 Hospital General Universitario Gregorio Marañón, Spain; 5 University of Cambridge, UK; 6 South London and Maudsley NHS Foundation Trust (SLaM), UK; 7 Universität Mannheim, Germany; 8 Radboud University Nijmegen Medical Center, The Netherlands; 9 Karakter Child and Adolescent Psychiatry University Center, The Netherlands

**Keywords:** anxiety, autism, depression, quality of life, well-being

## Abstract

Previous studies have reported reduced quality of life in autism. Improving quality of life for autistic people is, therefore, a key priority for clinical research and practice. However, the relative impact of core autism traits (e.g. social-communication difficulties), as compared to associated mental health symptoms (e.g. anxiety, depression) on quality of life remains poorly understood. This is despite at least 20%–50% of autistic individuals experiencing associated anxiety and/or depression symptoms. Hence, we measured subjective quality of life in 573 six to thirty-year-olds (autism spectrum disorder *N* = 344), using two widely validated questionnaires. Adults self-reported on the World Health Organization Quality of Life–Brief instrument. Parents of children/adolescents completed the Child Health and Illness Profile. We assessed individual variability across both measures and modelled associations between quality of life, core autism traits, anxiety, and depression symptoms. Across both age groups and quality of life measures, autistic individuals scored lower than comparison individuals, on average, particularly for physical health in adults (*d* = −1.24, 95% confidence interval: [−1.56, −0.93]) and school achievement for children/adolescents (*d* = −1.06, 95% confidence interval: [−1.29, −0.84]). However, a notable proportion of autistic individuals (36%–71% across quality of life domains) *did not* have reduced quality of life. Across ages and quality of life measures, severity of associated symptoms was significantly related to reduced quality of life on several domains, after accounting for core autism traits. Most notably, depression symptoms were related to reduced physical/psychological well-being in both adults (*β* ⩾ −0.34) and children/adolescents (*β* = −0.29, 95% confidence interval: [−0.36, −0.14]). For children/adolescents, anxiety symptoms (*β* ⩾ −0.28) and core social-communication difficulties (*β* ⩾ −0.22) were also related to subjective quality of life outcomes. Overall, findings indicate that not all autistic individuals experience reduced subjective quality of life. Variability in quality of life is significantly influenced by associated symptoms, across developmental stage. This may provide a tractable target for mental health services to improve quality of life for autistic individuals over the lifespan.

**Lay abstract:**

Previous studies suggest that some autistic individuals report lower satisfaction, or well-being, with different aspects of everyday life than those without autism. It is unclear whether this might be partly explained by symptoms of anxiety and/or depression, which affect at least 20%–50% of autistic people. In this study, we measured individual differences in well-being in 573 six to thirty-year-olds with and without a diagnosis of autism. We investigated whether individual differences in well-being were explained by autism traits (e.g. social-communication difficulties) and/or anxiety and depression symptoms. We showed that, though well-being was lower for some autistic individuals, compared to those without autism, many autistic individuals reported good well-being. Where well-being was reduced, this was particularly explained by depression symptoms, across all ages. For children/adolescents, anxiety and social-communication difficulties were also related to some aspects of well-being. Our study suggests that support and services for improving mental health, especially depression symptoms, may also improve broader outcomes for autistic people.

## Introduction

### Defining quality of life

Quality of life (QoL) is a fundamental outcome measure across psychiatry and healthcare, as recognised by the UK National Institute for Health and Care Excellence and World Health Organization (Mukuria et al., 2015; Provenzani et al., 2020; World Health Organization, 1998). Recently, QoL has also been identified as a gold standard for assessing well-being in autism – an outcome measure prioritised by autistic people and their families (McConachie et al., 2015). QoL can be defined as an individual’s satisfaction with their position in life, linked with their context, goals, expectations, standards and concerns (World Health Organization, 1998). Its multifaceted nature means that QoL cannot easily be reduced to a single score and must be considered across several domains, from physical and psychological health to social relationships. Subjective well-being may vary across these domains, each of them influenced by different contributing factors.

### QoL in autism

Accumulating evidence suggests that some autistic individuals experience reduced QoL, as compared to neurotypical individuals and those with other neurodevelopmental conditions, including attention deficit/hyperactivity disorder (ADHD; Barneveld et al., 2014; van Heijst & Geurts, 2015). This emphasises that improving QoL outcomes in autism is a key priority for clinical research and practice.

Nevertheless, it must be acknowledged that the experiences of autistic people vary substantially (Howlin & Moss, 2012). For example, while many individuals remain highly dependent on their families or support services into adulthood, others live independently – maintaining supportive relationships and regular employment (Howlin et al., 2004). Historically, studies that have highlighted such variability in the outcomes of autistic people have largely focused on objective QoL (see Henninger & Taylor, 2012 for a review). This means that they considered quantifiable, ‘normative’ markers of outcome, such as number of friendships, form of employment and contact with services (Billstedt et al., 2011; Bishop-Fitzpatrick et al., 2016; Steinhausen et al., 2016). The focus on objective QoL may be partly explained by the lack of available measurement tools for assessing subjective QoL in autism that are suitable for all ability levels and developmental stages (Ayres et al., 2018). However, the very definition of QoL emphasises its subjective nature, reflecting an individual’s perceptions of their well-being (i.e. how satisfied they are with their friendships, employment etc.; Coghill et al., 2009). Thus, there has been a more recent shift towards establishing individual variability in subjective QoL in autism and identifying the factors that may explain why a proportion of autistic individuals experience QoL reductions.

Existing research regarding subjective QoL outcomes would indicate that, on average, autistic people experience lower subjective well-being than neurotypical individuals, with a large effect size (*d* = −0.96; van Heijst & Geurts, 2015). This pattern of findings has been particularly reported for QoL domains indexing satisfaction with social relationships (e.g. feeling supported by friends/peers; Graham Holmes et al., 2020; Mason et al., 2018; Moss et al., 2017), psychological health (e.g. positive/negative affect, self-esteem, cognition; Hong et al., 2016) and – for autistic young people – school functioning (Kuhlthau et al., 2013). However, as with objective QoL outcomes, there may be substantial individual variability in the subjective well-being of each autistic person. This variability may also reflect the ‘person-environment fit’, or balance between objective (i.e. societal demands, expectations, accommodations) and subjective (i.e. individuals needs and preferences) outcomes (Billstedt et al., 2011; Henninger & Taylor, 2012).

### Factors associated with subjective QoL in autism

Notably, establishing the clinical determinants of subjective QoL reductions is essential for identifying where interventions and support would best be targeted to improve outcomes for some autistic people. Several studies have noted that higher severity of core autism traits, such as social-communication difficulties, are significantly associated with reduced subjective QoL in individuals with and without autism (Chiang & Wineman, 2014; de Vries & Geurts, 2015; Pisula et al., 2015; van Steensel et al., 2012). In addition, other factors highly implicated in autism, such as differences in executive functioning and sensory processing sensitivities, may also be related to QoL (de Vries & Geurts, 2015; Dijkhuis et al., 2016; Lin & Huang, 2019).

However, far fewer studies have investigated the impact of associated mental health symptoms on QoL in autism. This is despite the fact that ~20%–50% of autistic people experience associated symptoms of anxiety and/or depression (Hollocks et al., 2019; Lai et al., 2019; Simonoff et al., 2008). Furthermore, strong relationships have been identified between mental health symptoms and QoL in the wider population (Rapaport et al., 2005). Considering this, there has been a recent call for research based on well-characterised samples of individuals, varying in severity of neurodevelopmental/neuropsychiatric symptoms, to identify how QoL may be attributable to specific symptom dimensions that frequently co-occur (Jonsson et al., 2017).

Some existing research has begun to indicate relationships between associated mental health symptoms and facets of QoL in autism. For instance, elevated anxiety has been found to relate to increased physical health problems, including chronic gastrointestinal symptoms, in young people with autism (Mazurek et al., 2013). In longitudinal models, anxiety and depression symptoms early in development have also been linked to lower life satisfaction, greater social difficulties and reduced adaptive functioning by adulthood (Gotham et al., 2015). Furthermore, current mental health diagnoses are associated with *subjective* QoL in autistic adults, across domains of physical, psychological, social and environmental well-being, as rated with the World Health Organization Quality of Life–Brief instrument (WHOQoL-BREF; Mason et al., 2018). Generalised internalising problems are also associated with general subjective satisfaction and physical/psychological QoL during childhood, indexed with the Child Health and Illness Profile (Kuhlthau et al., 2013).

Nevertheless, we note some issues that require further investigation. First, previous studies have often utilised clinical or diagnostic cut-points to determine whether associated symptoms are absent or present – an approach which may underestimate the impact of these symptoms on QoL. Indeed, there are several diagnostic challenges to identifying co-occurring mental health/neurodevelopmental symptoms, meaning some individuals never receive a formal diagnosis and therefore remain underrepresented in such research (Hollocks et al., 2019; White et al., 2009). Second, where existing research has taken a dimensional approach to assess the impact of associated symptoms on QoL, across their full range of severity, other factors like core autism traits are not consistently controlled for in analyses (Adams et al., 2019) and/or internalising and emotional problems are considered as a general, unitary construct. This means that it is not possible to estimate the added impact of anxiety and/or depression symptoms in autism, over and above other factors known to influence QoL. Last, almost all existing studies have focused on single age groups, predominantly children (Bishop-Fitzpatrick et al., 2018), even though autism is a lifelong condition for which QoL reductions have been shown to persist into later adulthood (Graham Holmes et al., 2020; Mason et al., 2019; van Heijst & Geurts, 2015).

Hence, in this study, we aimed to investigate (1) individual variability in QoL among children, adolescents and adults on the autism spectrum, across several domains; and (2) their relationship with varying levels of core autism traits and associated symptoms of anxiety and depression. We addressed our aims using data from the European Autism Interventions Multicentre Study for Developing New Medications (EU-AIMS) Longitudinal European Autism Project (LEAP; Charman et al., 2017; Loth et al., 2017) – a well-characterised cohort of autistic males and females, diverse in age (6–30 years) and IQ (50–148). Based on the previous literature reported above, we predicted that autistic individuals would score lower for QoL than comparison individuals, but that we may also detect substantial individual variability in subjective QoL reports. In addition, since associated mental health symptoms have a strong impact on QoL in the wider population, we predicted that anxiety and/or depression symptoms would explain significant variance in QoL, even after accounting for core autism traits.

## Methods

### Participants

This study is based on data from the EU-AIMS LEAP cohort (please see Charman et al., 2017; Loth et al., 2017). A total of 573 males and females with and without a diagnosis of autism spectrum disorder (ASD *N* = 344), aged 6–30 years and with IQ of 50–148 and available QoL data were included. Participant characteristics and measures included in the current study are shown in Table 1.

**Table 1.** Descriptives and group comparisons for participant characteristics.

(a)	Adults (18–30 years**)**								
	ASD			Non-ASD			Group Comparison		
	*N*	*M* (*SD*)	Range	*N*	*M* (*SD*)	Range	$\chi_{\left(df,\;N\right)}^{2}$	*p*	*φ*
Sex: Males (Females)	72 (34)	–	–	56 (30)	–	–	0.17_(1, 192)_	0.68	0.03
							*Z*	*p*	*d* [95% CI]
Age (years)	106	23.08 (3.57)	18–30	86	23.32 (3.43)	18–30	0.64	0.52	−0.07 [−0.35, 0.22]
Full-scale IQ	106	105.64 (14.52)	75–148	86	109.70 (12.15)	85–142	1.86	0.06	−0.30 [−0.59, −0.01]
SRS-2 (Self)	104	64.58 (10.17)	40–89	76	46.83 (6.06)	37–67	9.93	<0.001***	2.05 [1.68, 2.41]
SSP	41	154.07 (26.33)	93–190	–	–	–	–	–	–
DAWBA Anxiety	96	3^a^	0–4	77	1^a^	0–4	7.11	<0.001***	1.28^b^
DAWBA Depression	84	1^a^	0–5	66	0^a^	0–3	3.19	0.001***	0.54^b^
DAWBA ADHD	59	0^a^	0–4	–	–	–	–	–	–
(b)	Children/Adolescents (6–17 years)								
	ASD			Non-ASD			Group Comparison		
	*N*	*M* (*SD*)	Range	*N*	*M* (*SD*)	Range	$\chi_{\left(df,\;N\right)}^{2}$	*p*	*φ*
Sex: Males (Females)	173 (65)	–	–	95 (48)	–	–	1.68_(1, 381)_	0.20	0.07
							*Z*	*p*	*d* [95% CI]
Age (years)	238	12.82 (3.11)	6–17	143	13.08 (3.13)	6–17	0.85	0.40	−0.08 [−0.29, 0.12]
Full-scale IQ	230	97.86 (19.55)	53–148^c^	141	103.56 (17.91)	50–140^c^	3.13	0.002***	−0.30 [−0.51, −0.09]
SRS-2 (Parent)	230	75.40 (10.71)	45–90	141	47.29 (8.35)	37–90	15.19	<0.001***	2.84 [2.55, 3.14]
SSP	186	133.62 (25.26)	69–189	116	176.32 (16.02)	75–190	12.53	<0.001***	−1.93 [−2.20, −1.65]
DAWBA Anxiety	198	2^a^	0–5	111	1^a^	0–4	9.88	<0.001***	1.35^b^
DAWBA Depression	193	1^a^	0–5	109	0^a^	0–2	5.42	<0.001***	0.65^b^
DAWBA ADHD	184	3^a^	0–5	105	0^a^	0–5	9.65	<0.001***	1.39^b^
(c)	Total Sample (6–30 years)								
	ASD			Non-ASD			Group Comparison		
	*N*	*M* (*SD*)	Range	*N*	*M* (*SD*)	Range	$\chi_{\left(df,\;N\right)}^{2}$	*p*	*φ*
Sex: Males (Females)	245 (99)	–	–	151 (78)	–	–	1.80_(1, 573)_	0.18	0.06
							*Z*	*p*	*d* [95% CI]
Age (years)	344	15.98 (5.75)	6–30	229	16.93 (5.93)	6–30	1.95	0.05*	−0.16 [−0.33, 0.00]
Full-scale IQ	336	100.31 (18.45)	54–148^c^	227	105.88 (16.22)	50–142^c^	3.85	<0.001***	−0.32 [−0.49, −0.15]
SRS-2 (Self)	168	63.11 (10.28)	40–89	133	47.02 (5.75)	37–67	12.38	<0.001***	1.88 [1.60, 2.15]
SRS-2 (Parent)	307	72.34 (11.91)	43–90	146	47.12 (8.26)	37–90	15.55	<0.001***	2.32 [2.07, 2.57]
SSP	227	137.31 (26.59)	69–190	120	176.63 (15.87)	75–190	12.49	<0.001***	−1.68 [−1.93, −1.42]
DAWBA Anxiety	294	3^a^	0–5	188	1^a^	0–4	11.95	<0.001***	1.28^b^
DAWBA Depression	277	1^a^	0–5	175	0^a^	0–3	6.14	<0.001***	0.61^b^
DAWBA ADHD	243	2^a^	0–5	105	0^a^	0–5	8.73	<0.001***	1.07^b^

ASD: autism spectrum disorder; CI: confidence interval; SRS-2: Social Responsiveness Scale–Second Edition; SSP: Short Sensory Profile; DAWBA: Development and Wellbeing Assessment; ADHD: attention deficit/hyperactivity disorder; *χ*^2^: chi-square statistic (degrees of freedom, sample size); *φ*: phi effect size for chi-square; *Z*: statistic for Mann–Whitney comparison; *d*: Cohen’s *d* effect size [95% confidence intervals].

a Median reported, due to ordinal nature of the scale.

b *r* effect size for Mann–Whitney *U* was converted to *d* using Rosenthal (1994).

c 51 individuals in the child/adolescent age range had IQ < 75 (ASD *N* = 37).

*p ⩽ 0.05; ***p ⩽ 0.002 (significant after Bonferroni correction; *p* = 0.05/28).

ASD and comparison groups did not significantly differ by sex ($\chi_{\left(1,\;573\right)}^{2}=1.80$, *p* = 0.18, *φ* = 0.06) and differed only nominally on age (*Z* = 1.95, *p* = 0.05, *d* = −0.16, 95% confidence interval (CI): [−0.33, 0.00]). Average IQ was significantly lower in the ASD group than the comparison group (*Z* = 3.85, *p* < 0.001, *d* = −0.32, 95% CI: [−0.49, −0.15]), though both ASD and comparison groups included individuals with mild intellectual disability (IQ ⩽ 75) in the child/adolescent (but not adult) age range. This study was approved by ethics committees at each participating site and informed consent/assent was obtained from all participants and their parents, where applicable.

### Materials and procedures

#### QoL measures

We administered two widely used QoL measures – in adults without intellectual disability, the 26-item self-report WHOQoL-BREF (The WHOQoL Group, 1996) and, for children and adolescents, the 45-item parent-report Child Health and Illness Profile–Child Edition (CHIP-CE; Riley et al., 2004).

The WHOQoL-BREF is currently one of the only QoL tools that has been validated for use with autistic adults without intellectual disability (Hong et al., 2016; McConachie et al., 2018). It assesses QoL across four domains: Physical Health (*To what extent do you feel that physical pain prevents you from doing what you need to do?*); Psychological Health (*How much do you enjoy your life?*); Social Relationships (*How satisfied are you with the support you get from your friends?*); and Environment (*To what extent do you have the opportunity for leisure activities?*). 106 autistic adults and 86 neurotypical adults, aged 18–30 years and with IQ > 75, completed the WHOQoL-BREF. Scores were transformed to a 0–100 scale for comparability with previous reports. Higher scores indicate better QoL. If <20% of values within a domain were missing (Physical Health *N* = 81, Psychological Health *N* = 82), we imputed them by taking the mean of non-missing values within that domain, as per the official scoring manual.

The CHIP-CE is a commonly administered parent-report tool that measures QoL across five domains: physical/psychological Comfort (*How often did your son/daughter have pain that really bothered him/her?*); Satisfaction (*How often does your son/daughter feel happy?*); Resilience (*How often does your son/daughter have an adult he/she can go to for help with a real problem?*); Risk Avoidance (*How often does your son/daughter do things that are dangerous?*); and Achievement (*How did he/she do in his/her schoolwork?*). We administered the CHIP-CE to parents of 381 children and adolescents – 146 aged 6–11 years (ASD *N* = 91) and 235 aged 12–17 years (ASD *N* = 147), including 51 individuals with IQ ⩽ 75 (ASD *N* = 37). We report mean scores, with higher scores reflecting better QoL. Imputation of missing values was not necessary for any domain.

#### Core autism traits

To measure the impact of current, core autism traits on QoL, we administered the Social Responsiveness Scale–Second Edition (SRS-2; Constantino & Gruber, 2012). We chose this measure because it has been validated for use across a wide age range (e.g. 6–30 years). Higher scores (sex-specific *T*-norms) indicate more severe difficulties. A self-report version was administered for all adults (i.e. ASD and comparison) aged 18–30 years and a parent-report version for all autistic individuals, as well as comparison individuals aged 6–17 years.

In the child/adolescent group, we also conducted supplementary analyses (Supplementary Table 3), using the Short Sensory Profile (SSP; Tomchek & Dunn, 2007) to index sensory processing differences commonly associated with autism that have been shown to relate to QoL. The SSP was completed by parents across all ages in the ASD group and ages 6–17 years in the comparison group, with lower scores indicating more sensory processing differences.

#### Associated symptom measures

Finally, we used the Development and Wellbeing Assessment (DAWBA; Goodman et al., 2000) to index anxiety and depression symptoms. We chose this measure because it assesses diagnostically relevant psychopathology and can be administered reliably to multiple informants (e.g. self and parent). Final scores result from the best available information – in *N* = 152, two informants (self and parent) were available, with the DAWBA providing a combined score, weighting both respondents’ answers. In *N* = 113 self-report and *N* = 215 parent-report only were available. Scores reflect symptom severity, ranked ordinally from 0 to 5. For anxiety, where multiple diagnoses were evaluated, we computed an overall score in accordance with Goodman et al. (2011), by deriving each participant’s highest score across individual anxiety disorders (separation anxiety, specific phobia, social phobia, generalised anxiety, panic disorder, agoraphobia, obsessive-compulsive disorder, post-traumatic stress disorder). Thus, the overall anxiety scale corresponds to the form of anxiety that the individual expresses most prominently.

In the supplement (Supplementary Table 3), we additionally included DAWBA-rated ADHD symptoms for the child/adolescent group, since ADHD symptoms have been shown to relate to QoL in autistic young people and children with a primary diagnosis of ADHD are reported to experience QoL reductions.

### Statistical analysis

Data were analysed using RStudio^®^, Version 3.5.1. As the DAWBA is rated on an ordinal scale, we used non-parametric statistics for group comparisons (Mann–Whitney) and correlations (Spearman’s *r_s_*). Bonferroni correction for multiple comparisons was applied throughout.

First, we assessed mean group differences in QoL scores between ASD and comparison groups, across each domain of the WHOQoL-BREF for adults aged 18–30 years and the CHIP-CE for children/adolescents aged 6–17 years, respectively. We then examined individual variability in QoL within the adult and child/adolescent subsamples from the ASD group, by calculating each individual’s score from the comparison group mean for each QoL domain. We used this criterion to quantify the proportion of autistic individuals scoring within or outside of 1 and 2 standard deviations from the comparison group mean.

Finally, we used linear regression models (‘*lm*’, function) to establish associations between QoL and core autism traits, anxiety and depression symptoms in the adult and child/adolescent subsamples from the ASD group. QoL domains from the WHOQoL-BREF for the adult sample and the CHIP-CE for the child/adolescent sample, respectively, were entered as dependent variables. Independent variables across all models were age, IQ, sex, core autism traits (SRS-2) and associated symptoms (DAWBA anxiety, depression). To be included in this analysis, individuals had to have available data for all variables in the regression model.

## Results

### Group comparisons on QoL

**Figure 1.**
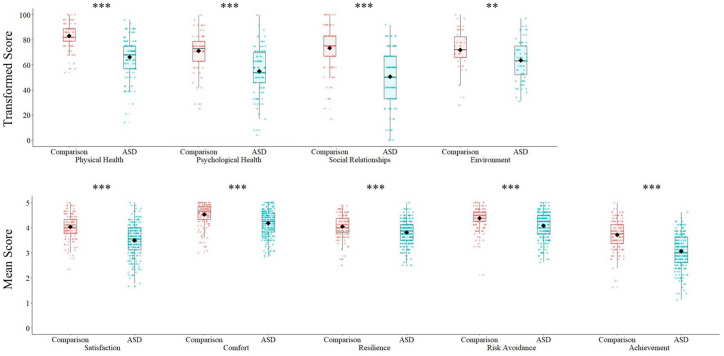
Boxplots showing group differences on QoL domains: (a) WHOQoL-BREF (adults 18–30 years); (b) CHIP-CE (children/adolescents 6–17 years). Individual data points are overlaid. The black diamond represents the mean. **p < 0.01; ***p < 0.006 (significant after Bonferroni correction; *p* = 0.05/9).

First, to establish whether results from the current sample replicated previous studies showing reduced QoL in autism, we compared average QoL scores for the ASD and comparison groups. Across both age groups, on both QoL measures, autistic individuals (as a group) scored significantly lower for QoL than comparison individuals (Table 2; Figure 1). Removing individuals with IQ < 75 (*N* = 51) from both ASD and comparison groups did not change the pattern of results, nor significance.

**Table 2.** Descriptives and group comparisons for QoL domains.

(a) WHOQoL-BREF (adults 18–30 years)									
	ASD			Comparison			*Z*	*p*	*d* [95% CI]
	*N*	*M* (*SD*)	Range	*N*	*M* (*SD*)	Range			
Physical Health	106	66.17 (16.00)	14–96	86	83.13 (9.96)	54–100	7.72	< 0.001***	−1.24 [−1.56, −0.93]
Psychological Health	106	55.09 (18.06)	4–100	86	71.38 (14.14)	25–100	6.69	< 0.001***	−0.99 [−1.29, −0.69]
Social Relationships	105	50.61 (21.34)	0–92	86	73.35 (17.43)	17–100	7.17	< 0.001***	−1.16 [−1.46, −0.85]
Environment	64	63.64 (15.92)	31–97	47	71.81 (15.59)	28–100	2.71	0.007**	−0.52 [−0.90, −0.14]
(b) CHIP-CE (Children/Adolescents 6–17 years)									
Satisfaction	238	3.50 (0.64)	1.67–5.00	143	4.04 (0.47)	2.33–5.00	8.25	< 0.001***	−0.93 [−1.15, −0.71]
Comfort	238	4.18 (0.50)	2.83–5.00	143	4.53 (0.43)	3.00–5.00	7.15	< 0.001***	−0.74 [−0.95, −0.52]
Resilience	238	3.82 (0.48)	2.50–5.00	143	4.05 (0.45)	2.50–4.88	4.57	0.006**	−0.49 [−0.70, −0.28]
Risk Avoidance	236	4.08 (0.52)	2.62–5.00	142	4.38 (0.42)	2.12–5.00	5.69	< 0.001***	−0.62 [−0.83, −0.41]
Achievement	223	3.06 (0.65)	1.12–4.62	140	3.72 (0.57)	1.62–5.00	8.92	< 0.001***	−1.06 [−1.29, −0.84]

ASD: autism spectrum disorder; CI: confidence interval; WHOQoL-BREF: World Health Organization Quality of Life–Brief instrument; CHIP-CE: Child Health and Illness Profile.

**p < 0.01, ***p < 0.006 (significant after Bonferroni correction; *p* = 0.05/9).

The strongest effect size for the difference in QoL between ASD and comparison adults was on the WHOQoL-BREF Physical Health domain (*Z* = 7.72, *p* < 0.001, *d* = −1.24, 95% CI: [−1.56, −0.93]). The strongest effect size for the difference between ASD and comparison children/adolescents was on the CHIP-CE school Achievement domain (*Z* = 8.92, *p* < 0.001, *d* = −1.06, 95% CI: [−1.29, −0.84]).

### Individual variability in QoL

**Figure 2.**
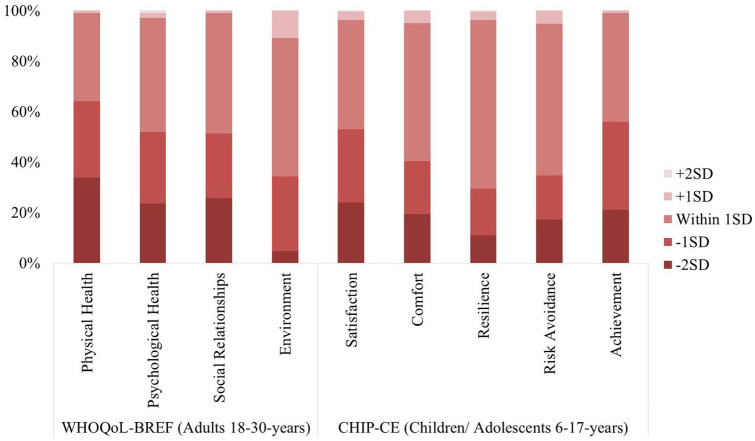
Stacked bar graph showing percentage of autistic individuals with QoL scores ±1 or 2 standard deviations from the comparison mean on each QoL domain of the WHOQoL-BREF and CHIP-CE, respectively.

Nevertheless, as highlighted by the individual data points presented in Figure 1, there was notable individual variability in QoL scores within both the ASD and comparison groups. Across WHOQoL-BREF domains, between 34.9% (Physical Health) and 54.7% (Environment) of autistic adults scored within 1 standard deviation of the comparison group mean (please see Figure 2). Similarly, across CHIP-CE domains, 43.0% (Achievement) to 66.8% (Resilience) of autistic children/adolescents scored within 1 standard deviation of the comparison group mean.

In contrast, some autistic individuals did have notable QoL reductions, scoring >2 standard deviations below the comparison group mean. Averaging across WHOQoL-BREF domains, this applied to 22.0% of autistic adults; and averaging across CHIP-CE domains, 18.5% of children/adolescents. Descriptives for the characteristics of individuals from the ASD group who scored within or above 1 standard deviation, as compared to those who scored below 1 standard deviation from the comparison group, are presented in Supplementary Table 2.

Of note, we confirmed that QoL scores in our comparison group corresponded to existing published norms (Hawthorne et al., 2006; Riley et al., 2001), except for CHIP-CE Comfort (comparison group scored higher than norms) and Achievement domains (lower than norms; Supplementary Table 1), indicating that our results were not driven by a particularly low- or high-scoring comparison group.

### The influence of core autism traits and associated anxiety/depression symptoms on QoL

Considering the significant individual variability in QoL identified in the previous section, we assessed how this variability may be related to severity of core autism traits and associated anxiety and depression symptoms, within the adult and child/adolescent subsamples from the ASD group. Regression coefficients and model fit statistics are shown in Table 3. Overall, regression models were significant for all WHOQoL-BREF and CHIP-CE domains, except WHOQoL Environment.

**Table 3.** Regression model fit and coefficients showing relationships within the autism group between WHOQoL-BREF domains for adults aged 18–30 years (*N* = 75) and CHIP-CE domains for children/adolescents aged 6–17 years (*N* = 180), with demographic factors, core autism traits and anxiety/depression symptoms.

		WHOQoL-BREF (adults 18–30 years)				
		Physical Health	Psychological Health	Social Relationships	Environment	–
		*β* [95% CI]	*β* [95% CI]	*β* [95% CI]	*β* [95% CI]	–
Demographic	Age	0.01 [−0.18, 0.21]	−0.04 [−0.23, 0.14]	−0.03 [−0.23, 0.18]	–	–
	IQ	−0.05 [−0.27, 0.15]	−0.17 [−0.37, 0.03]	0.002 [−0.22, 0.22]	–	–
	Sex	0.14 [−0.07, 0.36]	0.08 [−0.12, 0.29]	−0.15 [−0.39, 0.07]	–	–
Core traits	SRS-2 (Self)	−0.05 [−0.29, 0.19]	−0.26 [−0.49, −0.03]*	−0.23 [−0.50, 0.02]	–	–
Associated	DAWBA Anxiety^a^	−0.12 [−0.30, 0.10]	−0.07 [−0.25, 0.13]	0.04 [−0.18, 0.24]	–	–
	DAWBA Depression^a^	−0.38 [−0.40, −0.10]***	−0.34 [−0.37, −0.08]***	−0.40 [−0.44, −0.12]***	–	–
Model fit $\left(R_{adj}^{2}\right)$		*F*_(6, 75)_ = 4.39, *p* = 0.001***, $\eta_{p}^{2}=0.57$ (20.1%)	*F*_(6, 75)_ = 6.24, *p* < 0.001***, $\eta_{p}^{2}=0.60$ (28.0%)	*F*_(6, 74)_ = 3.62, *p* = 0.003***, $\eta_{p}^{2}=0.56$ (16.4%)	*F*_(6, 41)_ = 2.49, *p* = 0.04*, $\eta_{p}^{2}=0.58$ (15.9%)	–
		CHIP-CE (Children/Adolescents 6–17 years)				
		Satisfaction	Comfort	Resilience	Risk Avoidance	Achievement
		*β* [95% CI]	*β* [95% CI]	*β* [95% CI]	*β* [95% CI]	*β* [95% CI]
Demographic	Age	−0.13 [−0.29, −0.01]*	0.07 [−0.06, 0.20]	−0.23 [−0.39, −0.09]***	0.24 [0.11, 0.40]***	−0.002 [−0.14, 0.13]
	IQ	−0.06 [−0.20, 0.07]	0.05 [−0.07, 0.18]	0.07 [−0.07, 0.22]	0.06 [−0.09, 0.20]	0.34 [0.22, 0.50]***
	Sex	−0.005 [−0.15, 0.14]	0.07 [−0.06, 0.20]	−0.06 [−0.22, 0.09]	−0.18 [−0.36, −0.05]**	−0.16 [−0.32, −0.03]*
Core traits	SRS-2 (Parent)	−0.22 [−0.37, −0.08]***	−0.14 [−0.27, −0.01]*	−0.07 [−0.22, 0.08]	−0.19 [−0.34, −0.04]*	−0.34 [−0.48, −0.20]***
Associated	DAWBA Anxiety^a^	−0.28 [−0.34, −0.12]***	−0.33 [−0.35, −0.15]***	−0.01 [−0.12, 0.11]	0.03 [−0.09, 0.15]	0.003 [−0.11, 0.11]
	DAWBA Depression^a^	−0.28 [−0.38, −0.14]***	−0.29 [−0.36, −0.14]***	−0.22 [−0.32, −0.07]***	−0.18 [−0.29, −0.04]**	−0.15 [−0.24, −0.01]*
Model fit $\left(R_{adj}^{2}\right)$		*F*_(6, 180)_ = 13.69, *p* < 0.001***, $\eta_{p}^{2}=0.59$ (29.0%)	*F*_(6, 180)_ = 14.47, *p* < 0.001***, $\eta_{p}^{2}=0.60$ (30.3%)	*F*_(6, 180)_ = 4.45, *p* < 0.001***, $\eta_{p}^{2}=0.53$ (10.0%)	*F*_(6, 180)_ = 4.96, *p* < 0.001***, $\eta_{p}^{2}=0.54$ (11.3%)	*F*_(6, 169)_ = 11.74, *p* < 0.001***, $\eta_{p}^{2}=0.59$ (26.9%)

CI: confidence interval; SRS-2: Social Responsiveness Scale–Second Edition; *β*: standardised regression coefficient [95% confidence intervals]; *F: F* test for model significance (degrees of freedom, sample size); $\eta_{p}^{2}$: partial eta-squared effect size. Residuals from regression models were approximately normally distributed and collinearity diagnostics suggested no multicollinearity between variables.

a DAWBA scores were based on: Model a) multi-informant *N* = 58; self *N* = 107; parent *N* = 8; Model b) multi-informant *N* = 94; self *N* = 6; parent *N* = 207.

*p < 0.05, **p < 0.01, ***p < 0.008 (significant after Bonferroni correction; *p* = 0.05/6). The significance of bold values is ***p < 0.008.

#### WHOQoL-BREF: adults 18–30 years

For autistic adults, associated depression symptom severity was the only significant predictor of reduced QoL, after holding age, IQ, sex, core autism traits and anxiety symptoms constant. Significant associations between increasing depression symptoms and reduced QoL were apparent across WHOQoL-BREF domains of Physical Health (*β* = −0.38, *p* = 0.001, 95% CI for *β*: [−0.40, −0.10]), Psychological Health (*β* = −0.34, *p* = 0.002, 95% CI for *β*: [−0.37, −0.08]) and Social Relationships (*β* = −0.40, *p* < 0.001, 95% CI for *β*: [−0.44, −0.12]).

#### CHIP-CE: children/adolescents 6–17 years

Similar to findings from the adult group, in the child/adolescent sample, associated depression symptom severity was a significant predictor of reduced physical/psychological Comfort (*β* = −0.29, *p* < 0.001, 95% CI for *β*: [−0.36, −0.14]), after holding age, IQ, sex, core autism traits and anxiety symptoms constant. Anxiety symptoms were also significantly associated with reduced physical/psychological Comfort in this age group (*β* = −0.33, *p* < 0.001, 95% CI for *β*: [−0.35, −0.15]), holding other factors constant.

Furthermore, both anxiety (*β* = −0.28, *p* < 0.001, 95% CI for *β*: [−0.34, −0.12]) and depression symptoms (*β* = −0.28, *p* < 0.001, 95% CI for *β*: [−0.38, −0.14]) significantly contributed to decreased overall Satisfaction and depression symptoms to reduced Resilience (*β* = −0.22, *p* = 0.003, 95% CI for *β*: [−0.32, −0.07]).

In terms of core autism traits, higher SRS-2-rated difficulties were significantly related to decreased QoL on the CHIP-CE Satisfaction (*β* = −0.22, *p* < 0.001, 95% CI for *β*: [−0.37, −0.08]) and Achievement domains (*β* = −0.34, *p* < 0.001, 95% CI for *β*: [−0.48, −0.20]).

Finally, considering demographic factors, higher IQ was significantly related to higher scores on the Achievement domain (*β* = 0.34, *p* < 0.001, 95% CI for *β*: [0.22, 0.50]) and older age with decreased Resilience (*β* = −0.23, *p* = 0.002, 95% CI for *β*: [−0.39, −0.09]) but increased Risk Avoidance (*β* = 0.24, *p* < 0.001, 95% CI for *β*: [0.11, 0.40]). All these effects survived Bonferroni correction – additional, nominally significant results (*p* < 0.05 threshold) are flagged in Table 3. Supplementary analyses including SSP-rated sensory processing and DAWBA-rated ADHD symptoms for the child/adolescent group are presented in Supplementary Table 3.

## Discussion

### Individual variability in QoL in autism

This study is one of the first to demonstrate individual variability in QoL in autism, alongside group-level comparisons, across children, adolescents and adults, including those with mild intellectual disability. At the group level, QoL was significantly lower for autistic individuals, across all age groups, than comparison individuals. The area of QoL most reduced for autistic adults was physical health and, for children and adolescents, school achievement. Yet, analyses also revealed that, at the individual level, a notable proportion of autistic individuals in this study did *not* experience reduced QoL. In other words, many autistic individuals (36%–71% across QoL domains) reported, or were reported to have, a good QoL (according to thresholds on the subjective QoL measures utilised here) – emphasising the importance of accounting for subjective satisfaction when assessing outcomes in autism (Henninger & Taylor, 2012).

Our findings extend reports of individual variability in objective QoL in autism (Billstedt et al., 2011; Bishop-Fitzpatrick et al., 2016; Howlin et al., 2004), highlighting that variability is also prominent for subjective QoL. Therefore, group-level findings reported in QoL studies cannot be generalised to *all* autistic individuals. Moreover, understanding the strengths and protective factors that promote good QoL for some autistic individuals could inform best practice for improving QoL in those for whom it is reduced. For instance, access to social support and engaging in leisure activities and physical exercise have been shown to promote good QoL for some autistic people (Bishop-Fitzpatrick et al., 2017; Hamm & Yun, 2019; Mason et al., 2018; Renty & Roeyers, 2006). Overall, the investigation of individual differences in this study was aided by the multi-domain structure of the QoL instruments administered, which are invaluable for informing individualised intervention and support, since they identify what a good outcome means for each individual and the specific areas they may be struggling most (Coghill et al., 2009).

### Associated symptoms negatively impact QoL in autism, across development

Accordingly, though some autistic individuals report a QoL comparable to the majority of neurotypical individuals, it is important to understand the clinical determinants driving reduced QoL for those who do not. Results of this study showed that, across ages and different QoL measures (i.e. adults and children/adolescents; WHOQoL-BREF and CHIP-CE), higher severity of depression symptoms was significantly related to reduced QoL in individuals with ASD. Most notably, depression symptoms were strongly related to both physical and psychological well-being in adults (WHOQoL-BREF Physical/Psychological Health domains) and children/adolescents (CHIP-CE Comfort domain).

These findings are consistent with previous reports from general population samples, showing that depression symptoms are a key driver of QoL reductions (Rapaport et al., 2005; Roberts et al., 2014). In addition, it is noteworthy that depression symptoms were associated with physical, as well as psychological, well-being across age groups. Higher rates of physical health problems, such as epilepsy, gastrointestinal issues and inflammatory conditions, have been reported in ASD, as compared to the general population – likely underpinned by genetic and other biological factors (Bauman, 2010; Croen et al., 2015). Some physical health concerns commonly associated with autism, such as weight gain and sleep disturbance, may also be elevated as a side effect of medication use (Howes et al., 2018). Nonetheless, the increasingly acknowledged interconnection between mental and physical health indicates that mental health symptoms, like depression, may exacerbate physical health concerns and vice versa (Firth et al., 2019). Furthermore, mental health problems may present a barrier to accessing services (e.g. anxieties around healthcare settings, mental health perceived as ‘challenging behaviour’), increasing unmet healthcare needs (Doherty et al., 2020; Mason et al., 2018; Nicolaidis et al., 2013). Therefore, a targeted focus on improving mental health for autistic individuals may also aid in the wider management of physical health concerns commonly experienced in autism and individuals’ subjective satisfaction with their physical well-being.

Further to depression symptoms, in the child/adolescent group, we also identified relationships between anxiety and ADHD symptoms with QoL. First, increasing anxiety symptoms were significantly related to lower physical/psychological well-being and poorer overall satisfaction in this age group. Second, higher ADHD symptom severity was related to significantly reduced risk avoidance (Supplementary Table 3). It is possible that the relationships between associated symptoms and QoL reported here may be equally applicable to other neurodevelopmental conditions. For example, in accordance with the current report, a previous study of children with a primary diagnosis of ADHD showed that ADHD symptom severity was strongly, negatively related to risk avoidance and school achievement, also rated by the CHIP-CE (Coghill & Hodgkins, 2016). Thus, relationships between specific symptom dimensions and QoL outcomes may cross diagnostic boundaries – a hypothesis that requires future research including multiple diagnostic groups (e.g. ASD, anxiety/depressive disorders *and* ADHD) to be assessed in full. In addition, the fact that we did not identify associations between anxiety and QoL in the adult group in regression models, after holding other factors constant (in contrast to some previous reports; Lin & Huang, 2019; Smith et al., 2019), may further highlight the importance of considering the role and impact of depression symptoms for autistic adults. For instance, associations between anxiety and QoL could be partly mediated by co-occurring depression in some autistic adults, yet depression symptoms have rarely been assessed in previous reports.

As noted in the introductory section, previous studies that have also demonstrated significant associations between depression and QoL in autism have focused on individuals who meet diagnostic threshold for mental health symptom severity and/or are recruited from clinical settings (Mason et al., 2018, 2019; Park et al., 2019). The current findings demonstrate that symptoms which do not meet threshold for a clinical diagnosis can still have a notable impact on QoL, and therefore subtle/subthreshold indications for mental health in autistic individuals need to be routinely screened for and evaluated carefully.

### Core autism traits are associated with aspects of QoL in children/adolescents with autism

Aside from associated symptoms, there was some evidence for a relationship between core autism traits and QoL in this study, most prominently in children and adolescents. For instance, higher social-communication difficulties were associated with decreased overall satisfaction and school achievement – the QoL domain within which children/adolescents were also reported to experience the lowest well-being. This pattern of results was true after holding co-occurring anxiety and depression symptoms constant, which is important to note since social functioning can be impacted by mental health (e.g. anxiety/low mood leading to social difficulties; Cuve et al., 2018) and vice versa (e.g. social withdrawal leading to anticipatory anxiety/loneliness; Bellini, 2006; Hedley et al., 2018).

School settings are often large, complex social and sensory environments, which can be challenging for some young people on the autism spectrum. Many autistic young people face peer victimisation and bullying and report difficulties managing the pressures of the classroom environment (e.g. too fast-paced) and barriers for good communication with teachers (Sproston et al., 2017; van Roekel et al., 2010). Although around 71% of British children diagnosed with ASD attend mainstream school and spend most of their time in educational settings, there are currently few consistent, formal regulations in place to support them (Mandy et al., 2016; National Autistic Society, 2019). Furthermore, though there is increasing evidence for the potential effectiveness of school-based interventions to support autistic young people, many of these are yet to be translated to real-world practice (Anderson et al., 2017; Sutton et al., 2018). Hence, it is imperative to continue to develop strong research partnerships with schools to robustly evaluate the feasibility and effectiveness of diverse educational strategies for improving the QoL outcomes of young people on the autism spectrum.

For the adult group, we found less convincing evidence for relationships between core autism traits and QoL than in children/adolescents, overall. A simple explanation for this could be that the areas of QoL most affected by autism traits in our children/adolescent group (e.g. school achievement) are not reflected in the WHOQoL-BREF, or that the adult group did not include individuals with IQ < 75 (who may also present with more prominent autistic traits). However, it is also possible that some autistic individuals develop coping strategies for their difficulties associated with autism, but become more vulnerable to mental health problems across development. For instance, core autism traits generally become apparent within the first 2–5 years after birth. In contrast, associated symptoms, particularly depression, seem to emerge most commonly from late childhood and early adolescence (Ghaziuddin et al., 2002; Mayes et al., 2011; van Steensel et al., 2011). Indeed, clinical observations indicate that a proportion of autistic adults develop strategies to manage or ‘camouflage’ (i.e. mask) their difficulties associated with autism. In the long term, camouflaging requires high emotional and physical exertion – a previously reported risk factor for depression severity in autistic adults (Hull et al., 2017; Lai et al., 2017).

If valid, this interpretation implies that a decrease in severity of difficulties associated with autism should not be equated with QoL improvement, in the absence of direct QoL measurement. Indeed, intervention success is often assessed according to proximal outcomes (e.g. symptom reduction). However, more distal outcomes (e.g. QoL) tend to suggest fewer treatment gains in the areas of everyday functioning that may be particularly important to many autistic people and their families (Fletcher-Watson & McConachie, 2015; McConachie et al., 2015). For example, though cognitive behavioural therapy (CBT) is the most widely supported intervention approach for managing anxiety symptoms in autism (Kreslins et al., 2015; Sukhodolsky et al., 2013; White et al., 2018), improvements in QoL following CBT are not consistently identified (Flygare et al., 2020; van Steensel & Bogels, 2015).

Nevertheless, given that mental health symptoms in ASD can be modified by interventions, including CBT and mindfulness-based therapies (Gaigg et al., 2020), targeting these symptoms may hold potential for improving broader QoL outcomes. As research efforts to modify and develop novel interventions for improving mental health for autistic people evolve (see Cooper et al., 2018; Russell et al., 2019), our findings suggest that QoL is an informative outcome measure, beyond symptom severity. In further support of this, there is existing evidence that diverse outcomes measures in autism may have different contributing factors. For instance, previous data from the LEAP cohort have shown that parent-rated adaptive functioning (i.e. the ability to respond to ‘normative’ societal demands, such as socialisation with peers and everyday skills like washing/dressing) was predicted by core social-communication difficulties but not co-occurring mental health symptoms (Tillmann et al., 2019), whereas here we demonstrate that subjective QoL is strongly associated with mental health. These contrasting findings highlight the need for multiple measures of outcome in autism. Within such a framework, QoL tools have the potential to inform individualised approaches by indicating which outcomes are most meaningful for each person and the areas in which they may be experiencing most difficulties, thereby indicating primary intervention targets – for example, managing physical health problems or prioritising school-based support.

### Strengths and limitations

This study has a major strength in its heterogeneous sample of well over 500 males and females with and without autism, across developmental stage (6–30 years) and of a wide IQ range (50–148). These sample characteristics made it possible to establish that associated symptoms, particularly depression, are related to specific QoL outcomes in autism, across all developmental stages and levels of functioning. Nevertheless, we also note some limitations.

First, some authors have suggested that there may be overlap between measures of mental health symptoms and QoL measures (Coghill et al., 2009). In other words, it is possible that associations between anxiety and/or depression symptoms with psychological well-being were present because different questionnaire measures were indexing the same, or similar, experiences. However, from a theoretical perspective, the wide-ranging areas of everyday life and well-being that constitute QoL go beyond core psychiatric diagnostic criteria. In support of this, it should be noted that associations were apparent between depression symptoms and three out of four QoL domains of the WHOQoL-BREF in autistic adults, not just the psychological health domain. Similarly, depression symptoms were related to overall satisfaction in children/adolescents, in addition to physical/psychological health. This would suggest that the impact of depression symptoms on QoL in autism is more generalised, rather than specific to issues directly reflecting mental health.

A second limitation is that a self-report QoL measure was administered for adults without intellectual disability and a different parent-report measure for children and adolescents (including those with intellectual disability), meaning results from the adult and child/adolescent samples were not directly comparable. Previous research suggests that parents tend to rate the QoL of their son or daughter lower than their son or daughter would rate themselves (Hong et al., 2016; Jonsson et al., 2017) and different QoL measures use different item wordings for QoL domains. The lack of direct comparability of QoL measures across age groups in currently available QoL tools may reflect that priorities and concerns naturally evolve across development and particular outcomes (e.g. ability to complete schoolwork) are age-specific (Jonsson et al., 2017). Partly as a result of methodological challenges pertaining to age and development, there are currently few widely validated QoL measures for autistic individuals, particularly for those with mild intellectual disability and where novel measures are being developed, they are currently designed for specific age groups (e.g. adults only). The development of validated and robust QoL tools, accessible for individuals with difficulties in reading comprehension and/or speech and language, is thus an important area for future research (Ayres et al., 2018; McConachie et al., 2018). Despite this limitation, we identified a similar effect of depression symptom severity on physical and psychological well-being, across ages and different QoL measures. In addition, it was possible to collect self-report data from autistic adults with IQ > 75 in this study, further supporting that self-report tools are a valid method for assessing subjective QoL in ASD (Shipman et al., 2011).

## Conclusion

At the group level, average QoL ratings were significantly lower for autistic than comparison individuals, particularly for physical health in adults and school achievement in children and adolescents. This indicates that physical well-being and school support are key priorities for clinical research and practice, in the context of autism. Nevertheless, at the individual level, a notable proportion of autistic individuals reported a good QoL. Where QoL was reduced, this was most consistently accounted for by the severity of associated mental health symptoms, across age groups and QoL measures. In particular, associated depression symptoms impacted both physical and psychological well-being in children, adolescents and adults on the autism spectrum. In children and adolescents, anxiety, ADHD and core social-communication difficulties were also related to specific QoL outcomes. Taken together, these findings show that associated symptoms, particularly depression, must be specifically evaluated and targeted in order to improve the QoL outcomes of autistic people across development, using individualised approaches.

## Supplemental Material

Supplement_QoLAutism_cleanversion_17082020 – Supplemental material for How do core autism traits and associated symptoms relate to quality of life? Findings from the Longitudinal European Autism Project Click here for additional data file. Supplemental material, Supplement_QoLAutism_cleanversion_17082020 for How do core autism traits and associated symptoms relate to quality of life? Findings from the Longitudinal European Autism Project by Bethany FM Oakley, Julian Tillmann, Jumana Ahmad, Daisy Crawley, Antonia San José Cáceres, Rosemary Holt, Tony Charman, Tobias Banaschewski, Jan Buitelaar, Emily Simonoff, Declan Murphy and Eva Loth in Autism

## References

[bibr1-1362361320959959] Adams D. Clark M. Keen D. (2019). Using self-report to explore the relationship between anxiety and quality of life in children on the autism spectrum. Autism Research, 12(10), 1505–1515. 10.1002/aur.2155 31207183

[bibr2-1362361320959959] Anderson C. M. Smith T. Wilczynski S. M. (2017). Advances in school-based interventions for students with autism spectrum disorder: Introduction to the special issue. Behavior Modification, 42(1), 3–8. 10.1177/0145445517743582 29199446

[bibr3-1362361320959959] Ayres M. Parr J. R. Rodgers J. Mason D. Avery L. Flynn D. (2018). A systematic review of quality of life of adults on the autism spectrum. Autism, 22(7), 774–783. 10.1177/1362361317714988 28805071

[bibr4-1362361320959959] Barneveld P. S. Swaab H. Fagel S. van Engeland H. de Sonneville L. M. J. (2014). Quality of life: A case-controlled long-term follow-up study, comparing young high-functioning adults with autism spectrum disorders with adults with other psychiatric disorders diagnosed in childhood. Comprehensive Psychiatry, 55(2), 302–310. 10.1016/j.comppsych.2013.08.001 24290884

[bibr5-1362361320959959] Bauman M. L. (2010). Medical comorbidities in autism: Challenges to diagnosis and treatment. Neurotherapeutics, 7(3), 320–327. 10.1016/j.nurt.2010.06.001 20643385 PMC5084236

[bibr6-1362361320959959] Bellini S. (2006). The development of social anxiety in adolescents with autism spectrum disorders. Focus on Autism and Other Developmental Disabilities, 21(3), 138–145. 10.1177/10883576060210030201

[bibr7-1362361320959959] Billstedt E. Gillberg I. C. Gillberg C. (2011). Aspects of quality of life in adults diagnosed with autism in childhood: A population-based study. Autism, 15(1), 7–20. 10.1177/1362361309346066 20923888

[bibr8-1362361320959959] Bishop-Fitzpatrick L. Hong J. Smith L. E. Makuch R. A. Greenberg J. S. Mailick M. R. (2016). Characterizing objective quality of life and normative outcomes in adults with autism spectrum disorder: An exploratory latent class analysis. Journal of Autism and Developmental Disorders, 46(8), 2707–2719. 10.1007/s10803-016-2816-3 27207091 PMC5039043

[bibr9-1362361320959959] Bishop-Fitzpatrick L. Mazefsky C. A. Eack S. M. (2018). The combined impact of social support and perceived stress on quality of life in adults with autism spectrum disorder and without intellectual disability. Autism, 22(6), 703–711. 10.1177/1362361317703090 28666391 PMC5711618

[bibr10-1362361320959959] Bishop-Fitzpatrick L. Smith DaWalt L. Greenberg J. S. Mailick M. R. (2017). Participation in recreational activities buffers the impact of perceived stress on quality of life in adults with autism spectrum disorder. Autism Research, 10(5), 973–982. 10.1002/aur.1753 28244233 PMC5588899

[bibr11-1362361320959959] Charman T. Loth E. Tillmann J. Crawley D. Wooldridge C. Goyard D. … Buitelaar J. K. (2017). The EU-AIMS Longitudinal European Autism Project (LEAP): Clinical characterisation. Molecular Autism, 8(1), 1–21. 10.1186/s13229-017-0145-9 28649313 PMC5481972

[bibr12-1362361320959959] Chiang H. M. Wineman I. (2014). Factors associated with quality of life in individuals with autism spectrum disorders: A review of literature. Research in Autism Spectrum Disorders, 8(8), 974–986. 10.1016/j.rasd.2014.05.003

[bibr13-1362361320959959] Coghill D. Danckaerts M. Sonuga-Barke E. Sergeant J. (2009). Practitioner Review: Quality of life in child mental health – Conceptual challenges and practical choices. Journal of Child Psychology and Psychiatry and Allied Disciplines, 50(5), 544–561. 10.1111/j.1469-7610.2009.02008.x 19432681

[bibr14-1362361320959959] Coghill D. Hodgkins P. (2016). Health-related quality of life of children with attention-deficit/hyperactivity disorder versus children with diabetes and healthy controls. European Child & Adolescent Psychiatry, 25(3), 261–271. 10.1007/s00787-015-0728-y 26054300 PMC4769721

[bibr15-1362361320959959] Constantino J. Gruber C. (2012). Social Responsiveness Scale–Second Edition (SRS-2). Western Psychological Services.

[bibr16-1362361320959959] Cooper K. Loades M. E. Russell A. (2018). Adapting psychological therapies for autism. Research in Autism Spectrum Disorders, 45, 43–50. 10.1016/j.rasd.2017.11.002 30245739 PMC6150418

[bibr17-1362361320959959] Croen L. A. Zerbo O. Qian Y. Massolo M. L. Rich S. Sidney S. Kripke C. (2015). The health status of adults on the autism spectrum. Autism, 19(7), 814–823. 10.1177/1362361315577517 25911091

[bibr18-1362361320959959] Cuve H. C. Gao Y. Fuse A. (2018). Is it avoidance or hypoarousal? A systematic review of emotion recognition, eye-tracking, and psychophysiological studies in young adults with autism spectrum conditions. Research in Autism Spectrum Disorders, 55, 1–13. 10.1016/j.rasd.2018.07.002

[bibr19-1362361320959959] de Vries M. Geurts H (2015). Influence of autism traits and executive functioning on quality of life in children with an autism spectrum disorder. Journal of Autism and Developmental Disorders, 45(9), 2734–2743. 10.1007/s10803-015-2438-1 25835211 PMC4553152

[bibr20-1362361320959959] Dijkhuis R. R. Ziermans T. B. Van Rijn S. Staal W. G. Swaab H. (2016). Self-regulation and quality of life in high-functioning young adults with autism. Autism, 21(7), 896–906. 10.1177/1362361316655525 27407040 PMC5625847

[bibr21-1362361320959959] Doherty M. Sullivan J. D. Neilson S. D. (2020). Barriers to healthcare for autistic adults: Consequences & policy implications. A cross-sectional study. MedRxiv. 10.1101/2020.04.01.20050336

[bibr22-1362361320959959] Firth J. Siddiqi N. Koyanagi A. Siskind D. Rosenbaum S. Galletly C. … Stubbs B. (2019). The Lancet Psychiatry Commission: A blueprint for protecting physical health in people with mental illness. The Lancet Psychiatry, 6(8), 675–712. 10.1016/S2215-0366(19)30132-4 31324560

[bibr23-1362361320959959] Fletcher-Watson S. McConachie H. (2015). The search for an early intervention outcome measurement tool in autism. Focus on Autism and Other Developmental Disabilities, 32(1), 71–80. 10.1177/1088357615583468

[bibr24-1362361320959959] Flygare O. Andersson E. Ringberg H. Hellstadius A.-C. Edbacken J. Enander J. … Rück C. (2020). Adapted cognitive behavior therapy for obsessive–compulsive disorder with co-occurring autism spectrum disorder: A clinical effectiveness study. Autism, 24(1), 190–199. 10.1177/1362361319856974 31187645

[bibr25-1362361320959959] Gaigg S. B. Flaxman P. E. McLaven G. Shah R. Bowler D. M. Meyer B. … South M. (2020). Self-guided mindfulness and cognitive behavioural practices reduce anxiety in autistic adults: A pilot 8-month waitlist-controlled trial of widely available online tools. Autism, 24, 867–883. 10.1177/1362361320909184 32267168 PMC7418273

[bibr26-1362361320959959] Ghaziuddin M. Ghaziuddin N. Greden J. (2002). Depression in persons with autism: Implications for research and clinical care. Journal of Autism and Developmental Disorders, 32(4), 299–306. 10.1023/A:1016330802348 12199134

[bibr27-1362361320959959] Goodman A. Heiervang E. Collishaw S. Goodman R. (2011). The ‘DAWBA bands’ as an ordered-categorical measure of child mental health: Description and validation in British and Norwegian samples. Social Psychiatry and Psychiatric Epidemiology, 46(6), 521–532. 10.1007/s00127-010-0219-x 20376427

[bibr28-1362361320959959] Goodman R. Ford T. Richards H. Gatward R. Meltzer H. (2000). The development and well-being assessment: Description and initial validation of an integrated assessment of child and adolescent psychopathology. Journal of Child Psychology and Psychiatry, 41(5), 645–655. 10.1111/j.1469-7610.2000.tb02345.x 10946756

[bibr29-1362361320959959] Gotham K. Brunwasser S. M. Lord C. (2015). Depressive and anxiety symptom trajectories from school age through young adulthood in samples with autism spectrum disorder and developmental delay. Journal of the American Academy of Child and Adolescent Psychiatry, 54(5), 369–373. e3. 10.1016/j.jaac.2015.02.005 25901773 PMC4407021

[bibr30-1362361320959959] Graham Holmes L. Zampella C. J. Clements C. McCleery J. P. Maddox B. B. Parish-Morris J. … Miller J. S (2020). A lifespan approach to patient-reported outcomes and quality of life for people on the autism spectrum. Autism Research, 13, 970–987. 10.1002/aur.2275 32154664

[bibr31-1362361320959959] Hamm J. Yun J. (2019). Influence of physical activity on the health-related quality of life of young adults with and without autism spectrum disorder. Disability and Rehabilitation, 41(7), 763–769. 10.1080/09638288.2017.1408708 29182033

[bibr32-1362361320959959] Hawthorne G. Herrman H. Murphy B. (2006). Interpreting the WHOQOL-Brèf: Preliminary population norms and effect sizes. Social Indicators Research, 77(1), 37–59. 10.1007/s11205-005-5552-1

[bibr33-1362361320959959] Hedley D. Uljarević M. Foley K.-R. Richdale A. Trollor J. (2018). Risk and protective factors underlying depression and suicidal ideation in Autism Spectrum Disorder. Depression and Anxiety, 35(7), 648–657. 10.1002/da.22759 29659141

[bibr34-1362361320959959] Henninger N. A. Taylor J. L. (2012). Outcomes in adults with autism spectrum disorders: A historical perspective. Autism, 17(1), 103–116. 10.1177/1362361312441266 22914775 PMC3769945

[bibr35-1362361320959959] Hollocks M. J. Lerh J. W. Magiati I. Meiser-Stedman R. Brugha T. S. (2019). Anxiety and depression in adults with autism spectrum disorder: A systematic review and meta-analysis. Psychological Medicine, 49(4), 559–572. 10.1017/S0033291718002283 30178724

[bibr36-1362361320959959] Hong J. Bishop-Fitzpatrick L. Smith L. E. Greenberg J. S. Mailick M. R. (2016). Factors associated with subjective quality of life of adults with autism spectrum disorder: Self-report versus maternal reports. Journal of Autism and Developmental Disorders, 46(4), 1368–1378. 10.1007/s10803-015-2678-0 26707626 PMC4788526

[bibr37-1362361320959959] Howes O. D. Rogdaki M. Findon J. L. Wichers R. H. Charman T. King B. H. … Murphy D. G. (2018). Autism spectrum disorder: Consensus guidelines on assessment, treatment and research from the British Association for Psychopharmacology. Journal of Psychopharmacology, 32(1), 3–29. 10.1177/0269881117741766 29237331 PMC5805024

[bibr38-1362361320959959] Howlin P. Goode S. Hutton J. Rutter M. (2004). Adult outcome for children with autism. Journal of Child Psychology and Psychiatry, 45(2), 212–229. 10.1111/j.1469-7610.2004.00215.x 14982237

[bibr39-1362361320959959] Howlin P. Moss P. (2012). Adults with autism spectrum disorders. Canadian Journal of Psychiatry, 57(5), 275–283. 10.1177/070674371205700502 22546059

[bibr40-1362361320959959] Hull L. Petrides K. V. Allison C. Smith P. Baron-Cohen S. Lai M. C. Mandy W. (2017). ‘Putting on my best normal’: Social camouflaging in adults with autism spectrum conditions. Journal of Autism and Developmental Disorders, 47(8), 2519–2534. 10.1007/s10803-017-3166-5 28527095 PMC5509825

[bibr41-1362361320959959] Jonsson U. Alaie I. Löfgren Wilteus A. Zander E. Marschik P. B. Coghill D. Bölte S. (2017). Annual Research Review: Quality of life and childhood mental and behavioural disorders – A critical review of the research. Journal of Child Psychology and Psychiatry and Allied Disciplines, 58(4), 439–469. 10.1111/jcpp.12645 27709604

[bibr42-1362361320959959] Kreslins A. Robertson A. E. Melville C. (2015). The effectiveness of psychosocial interventions for anxiety in children and adolescents with autism spectrum disorder: A systematic review and meta-analysis. Child and Adolescent Psychiatry and Mental Health, 9, Article 22 10.1186/s13034-015-0054-7 26120361 PMC4482189

[bibr43-1362361320959959] Kuhlthau K. Kovacs E. Hall T. Clemmons T. Orlich F. Delahaye J. Sikora D. (2013). Health-related quality of life for children with ASD: Associations with behavioral characteristics. Research in Autism Spectrum Disorders, 7(9), 1035–1042. 10.1016/j.rasd.2013.04.006

[bibr44-1362361320959959] Lai M.-C. Kassee C. Besney R. Bonato S. Hull L. Mandy W. … Ameis S. H. (2019). Prevalence of co-occurring mental health diagnoses in the autism population: A systematic review and meta-analysis. The Lancet Psychiatry, 6(10), 819–829. 10.1016/S2215-0366(19)30289-5 31447415

[bibr45-1362361320959959] Lai M. C. Lombardo M. V. Ruigrok A. N. V. Chakrabarti B. Auyeung B. Szatmari P. … Baron-Cohen S. (2017). Quantifying and exploring camouflaging in men and women with autism. Autism, 21(6), 690–702. 10.1177/1362361316671012 27899710 PMC5536256

[bibr46-1362361320959959] Lin L.-Y. Huang P.-C. (2019). Quality of life and its related factors for adults with autism spectrum disorder. Disability and Rehabilitation, 41(8), 896–903. 10.1080/09638288.2017.1414887 29228834

[bibr47-1362361320959959] Loth E. Charman T. Mason L. Tillmann J. Jones E. Wooldridge, C., . . . Buitelaar, J. (2017). The EU-AIMS Longitudinal European Autism Project (LEAP): Design and methodologies to identify and validate stratification biomarkers for autism spectrum disorders. Molecular Autism, 8, Article 24 10.1186/s13229-017-0146-8 28649312 PMC5481887

[bibr48-1362361320959959] Mandy W. Murin M. Baykaner O. Staunton S. Hellriegel J. Anderson S. Skuse D. (2016). The transition from primary to secondary school in mainstream education for children with autism spectrum disorder. Autism, 20(1), 5–13. 10.1177/1362361314562616 25576142 PMC4702244

[bibr49-1362361320959959] Mason D. Mackintosh J. McConachie H. Rodgers J. Finch T. Parr J. R. (2019). Quality of life for older autistic people: The impact of mental health difficulties. Research in Autism Spectrum Disorders, 63, 13–22. 10.1016/j.rasd.2019.02.007

[bibr50-1362361320959959] Mason D. McConachie H. Garland D. Petrou A. Rodgers J. Parr J. R. (2018). Predictors of quality of life for autistic adults. Autism Research, 11, 1138–1147. 10.1002/aur.1965 29734506 PMC6220831

[bibr51-1362361320959959] Mayes S. D. Calhoun S. L. Murray M. J. Ahuja M. Smith L. A. (2011). Anxiety, depression, and irritability in children with autism relative to other neuropsychiatric disorders and typical development. Research in Autism Spectrum Disorders, 5(1), 474–485. 10.1016/j.rasd.2010.06.012

[bibr52-1362361320959959] Mazurek M. O. Vasa R. A. Kalb L. G. Kanne S. M. Rosenberg D. Keefer A. … Lowery L. A. (2013). Anxiety, sensory over-responsivity, and gastrointestinal problems in children with autism spectrum disorders. Journal of Abnormal Child Psychology, 41(1), 165–176. 10.1007/s10802-012-9668-x 22850932

[bibr53-1362361320959959] McConachie H. Mason D. Parr J. R. Garland D. Wilson C. Rodgers J. (2018). Enhancing the validity of a quality of life measure for autistic people. Journal of Autism and Developmental Disorders, 48(5), 1596–1611. 10.1007/s10803-017-3402-z 29188584 PMC5889785

[bibr54-1362361320959959] McConachie H. Parr J. R. Glod M. Hanratty J. Livingstone N. Oono I. P. … Williams K. (2015). Systematic review of tools to measure outcomes for young children with autism spectrum disorder. Health Technology Assessment, 19(41), 1–506. 10.3310/hta19410 PMC4781156 26065374

[bibr55-1362361320959959] Moss P. Mandy W. Howlin P. (2017). Child and adult factors related to quality of life in adults with autism. Journal of Autism and Developmental Disorders, 47(6), 1830–1837. 10.1007/s10803-017-3105-5 28343343 PMC5432629

[bibr56-1362361320959959] Mukuria C. Connell J. Carlton J. Peasgood T. Scope A. Jones K. Brazier J. (2015). Developing content for a new generic QALY measure: Results from a qualitative literature review (E-QALY Project). Value in Health, 13(6): pS110.

[bibr57-1362361320959959] National Autistic Society. (2019). The Autism Act, 10 Years On: A report from the All Party Parliamentary Group on Autism on understanding, services and support for autistic people and their families in England. https://pearsfoundation.org.uk/wp-content/uploads/2019/09/APPGA-Autism-Act-Inquiry-Report.pdf

[bibr58-1362361320959959] Nicolaidis C. Raymaker D. McDonald K. Dern S. Boisclair W. C. Ashkenazy E. Baggs A. (2013). Comparison of healthcare experiences in autistic and non-autistic adults: A cross-sectional online survey facilitated by an academic-community partnership. Journal of General Internal Medicine, 28(6), 761–769. 10.1007/s11606-012-2262-7 23179969 PMC3663938

[bibr59-1362361320959959] Park S. H. Song Y. J. C. Demetriou E. A. Pepper K. L. Norton A. Thomas E. E. … Guastella A. J. (2019). Disability, functioning, and quality of life among treatment-seeking young autistic adults and its relation to depression, anxiety, and stress. Autism, 23(7), 1675–1686. 10.1177/1362361318823925 30654629

[bibr60-1362361320959959] Pisula E. Danielewicz D. Kawa R. Pisula W. (2015). Autism spectrum quotient, coping with stress and quality of life in a non–clinical sample – an exploratory report. Health and Quality of Life Outcomes, 13, Article 173 10.1186/s12955-015-0370-x 26503411 PMC4624179

[bibr61-1362361320959959] Provenzani U. Fusar-Poli L. Brondino N. Damiani S. Vercesi M. Meyer N. … Politi P. (2020). What are we targeting when we treat autism spectrum disorder? A systematic review of 406 clinical trials. Autism, 24, 274–284. 10.1177/1362361319854641 31269800

[bibr62-1362361320959959] Rapaport M. H. Clary C. Fayyad R. Endicott J. (2005). Quality-of-life in depressive and anxiety disorders. American Journal of Psychiatry, 162(6), 1171–1178. 10.1176/appi.ajp.162.6.1171 15930066

[bibr63-1362361320959959] Renty J. O. Roeyers H. (2006). Quality of life in high-functioning adults with autism spectrum disorder: The predictive value of disability and support characteristics. Autism, 10(5), 511–524. 10.1177/1362361306066604 16940316

[bibr64-1362361320959959] Riley A. W. Forrest C. B. Starfield B. Rebok G. W. Robertson J. A. Green B. F. (2004). The parent report form of the CHIP-child edition: Reliability and validity. Medical Care, 42(3), 210–220. 10.1097/01.mlr.0000114909.33878.ca 15076820

[bibr65-1362361320959959] Riley A. W. Robsertson J. A. Forrest C. B. Al E. (2001). Technical manual for the Child Health and Illness Profile-Child Edition (CHIP-CETM) Parent and Child Report Forms. John Hopkins University.

[bibr66-1362361320959959] Roberts J. Lenton P. Keetharuth A. D. Brazier J. (2014). Quality of life impact of mental health conditions in England: Results from the adult psychiatric morbidity surveys. Health and Quality of Life Outcomes, 12(1), 1–10. 10.1186/1477-7525-12-6 24422899 PMC3901021

[bibr67-1362361320959959] Rosenthal R. (1994). Parametric measures of effect size. In Cooper H. Hedges L. V. (Eds.), The handbook of research synthesis (pp. 231–244). Russell Sage Foundation.

[bibr68-1362361320959959] Russell A. Gaunt D. Cooper K. Horwood J. Barton S. Ensum I. … Wiles N. (2019). Guided self-help for depression in autistic adults: The ADEPT feasibility RCT. Health Technology Assessment, 23, 1–94. 10.3310/hta23680 PMC6943380 31856942

[bibr69-1362361320959959] Shipman D. L. Sheldrick R. C. Perrin E. C. (2011). Quality of life in adolescents with autism spectrum disorders: Reliability and validity of self-reports. Journal of Developmental and Behavioral Pediatrics, 32(2), 85–89. 10.1097/DBP.0b013e318203e558 21187785

[bibr70-1362361320959959] Simonoff E. Pickles A. Charman T. Chandler S. Loucas T. Baird G. (2008). Psychiatric disorders in children with autism spectrum disorders: Prevalence, comorbidity, and associated factors in a population-derived sample. Journal of the American Academy of Child and Adolescent Psychiatry, 47(8), 921–929. 10.1097/CHI.0b013e318179964f 18645422

[bibr71-1362361320959959] Smith I. C. Ollendick T. H. White S. W. (2019). Anxiety moderates the influence of ASD severity on quality of life in adults with ASD. Research in Autism Spectrum Disorders, 62, 39–47. 10.1016/j.rasd.2019.03.001

[bibr72-1362361320959959] Sproston K. Sedgewick F. Crane L. (2017). Autistic girls and school exclusion: Perspectives of students and their parents. Autism & Developmental Language Impairments, 2, 2396941517706172 10.1177/2396941517706172

[bibr73-1362361320959959] Steinhausen H.-C. Mohr Jensen C. Lauritsen M. B. (2016). A systematic review and meta-analysis of the long-term overall outcome of autism spectrum disorders in adolescence and adulthood. Acta Psychiatrica Scandinavica, 133(6), 445–452. 10.1111/acps.12559 26763353

[bibr74-1362361320959959] Sukhodolsky D. G. Bloch M. H. Panza K. E. Reichow B. (2013). Cognitive-behavioral therapy for anxiety in children with high-functioning autism: A meta-analysis. Pediatrics, 132(5), e1341–e1350. 10.1542/peds.2013-1193 24167175 PMC3813396

[bibr75-1362361320959959] Sutton B. M. Webster A. A. Westerveld M. F. (2018). A systematic review of school-based interventions targeting social communication behaviors for students with autism. Autism, 23(2), 274–286. 10.1177/1362361317753564 29382208

[bibr76-1362361320959959] Tillmann J. San Jose Caceres A. Chatham C. H. Crawley D. Holt R. Oakley B. … Charman T. (2019). Investigating the factors underlying adaptive functioning in autism in the EU-AIMS Longitudinal European Autism Project. Autism Research, 12(4), 645–657. 10.1002/aur.2081 30741482 PMC6519242

[bibr77-1362361320959959] Tomchek S. D. Dunn W. (2007). Sensory processing in children with and without autism: A comparative study using the Short Sensory Profile. American Journal of Occupational Therapy, 61(2), 190–200. 10.5014/ajot.61.2.190 17436841

[bibr78-1362361320959959] van Heijst B. F. C. Geurts H. M (2015). Quality of life in autism across the lifespan: A meta-analysis. Autism, 19(2), 158–167. 10.1177/1362361313517053 24443331

[bibr79-1362361320959959] van Roekel E. Scholte R. H. J. Didden R (2010). Bullying among adolescents with autism spectrum disorders: Prevalence and perception. Journal of Autism and Developmental Disorders, 40(1), 63–73. 10.1007/s10803-009-0832-2 19669402 PMC2809311

[bibr80-1362361320959959] van Steensel F. J. A. Bogels S. M (2015). CBT for anxiety disorders in children with and without autism spectrum disorders. Journal of Consulting and Clinical Psychology, 83(3), 512–523. 10.1037/a0039108 25894668

[bibr81-1362361320959959] van Steensel F. J. A. Bögels S. M. Dirksen C. D (2012). Anxiety and quality of life: clinically anxious children with and without autism spectrum disorders compared. Journal of Clinical Child & Adolescent Psychology, 41(6), 731–738. 10.1080/15374416.2012.698725 22775580

[bibr82-1362361320959959] van Steensel F. J. A. Bögels S. M. Perrin S (2011). Anxiety disorders in children and adolescents with autistic spectrum disorders: A meta-analysis. Clinical Child and Family Psychology Review, 14(3), 302–317. 10.1007/s10567-011-0097-0 21735077 PMC3162631

[bibr83-1362361320959959] White S. Oswald D. Ollendick T. Scahill L. (2009). Anxiety in children and adolescents with autism spectrum disorders. Clinical Psychology Review, 29(3), 216–229. 10.1016/j.cpr.2009.01.003.Anxiety 19223098 PMC2692135

[bibr84-1362361320959959] White S. W. Simmons G. L. Gotham K. O. Conner C. M. Smith I. C. Beck K. B. Mazefsky C. A. (2018). Psychosocial treatments targeting anxiety and depression in adolescents and adults on the autism spectrum: Review of the latest research and recommended future directions. Current Psychiatry Reports, 20(10), Article 82 10.1007/s11920-018-0949-0 30155584 PMC6421847

[bibr85-1362361320959959] The WHOQoL Group. (1996, 12). WHOQoL-BREF: Introduction, Administration, Scoring and Generic Version of the Assessment. Programme on Mental Health 10.1037/t01408-000

[bibr86-1362361320959959] World Health Organization. (1998). WHOQOL: Measuring quality of life. Psychological Medicine, 28(3), 551–558. https://doi.org/10.5.12 9626712 10.1017/s0033291798006667

