# Supplementary material for: How do core autism traits and associated symptoms relate to quality of life? Findings from the Longitudinal European Autism Project
Source: Autism. 2020 Oct 7;25(2):389–404. doi: 10.1177/1362361320959959 (PMC7874383; doi:10.1177/1362361320959959)
Supplement: Supplement_QoLAutism_cleanversion_17082020 – Supplemental material for How do core autism traits and associated symptoms relate to quality of life? Findings from the Longitudinal European Autism Project [file Supplement_QoLAutism_cleanversion_17082020.pdf]

Supplementary Table 1.

Effect sizes for differences between published scores on the WHOQoL-BREF (20-to-29-years; Hawthorne, Herrman, & Murphy, 2006)/ CHIP-CE (John Hopkins University, 2001) and our comparison group.

		**Published Norms**	**Comparison Group**	***d* [95% CI]**
**WHOQoL-BREF *M* (*SD*)**	**Physical Health**	85.40 (10.90)	83.13 (9.96)	0.22 [-0.14, 0.58]
	**Psychological Health**	71.40 (17.50)	71.38 (14.14)	0.001 [-0.35, 0.36]
	**Social Relationships**	72.90 (18.80)	73.35 (17.43)	-0.03 [-0.38, 0.33]
	**Environment**	74.30 (14.00)	71.81 (15.59)	0.17 [-0.24, 0.57]
**CHIP-CE *M* (*SD*)**	**Satisfaction**	4.26 (0.64)	4.04 (0.47)	0.35 [0.22, 0.49]
	**Comfort**	3.93 (0.70)	4.53 (0.43)	-0.89 [-1.03, -0.75]
	**Resilience**	3.97 (0.70)	4.05 (0.45)	-0.12 [-0.25, 0.02]
	**Risk Avoidance**	4.37 (0.67)	4.38 (0.42)	-0.02 [-0.15, 0.12]
	**Achievement**	4.22 (0.63)	3.72 (0.57)	0.80 [0.66, 0.94]

Note: WHOQoL-BREF=World Health Organisation Quality of Life – Brief instrument; CHIP-CE=Child Health and Illness Profile; *d*=Cohen's *d* effect size [95% confidence intervals].

Supplementary Table 2.

Descriptives and group comparisons for autistic individuals who scored within/ above, as compared to below, 1 standard deviation from the comparison group mean across domains from: a) WHOQoL-BREF for adults 18-30-years, and; b) CHIP-CE for children/ adolescents 6-17-years.

a) WHOQoL-BREF (Adults 18-30-years)

	**Physical Health**			**Psychological Health**			**Social Relationships**			**Environment**			**-**
	**>1SD below**	**Within/ above 1SD**	***d* [95% CI]**	**>1SD below**	**Within/ above 1SD**	***d* [95% CI]**	**>1SD below**	**Within/ above 1SD**	***d* [95% CI]**	**>1SD below**	**Within/ above 1SD**	***d* [95% CI]**	**-**
N	68	38	-	55	51	-	54	51	-	22	42	-	-
**Sex: Males (Females)**	43 (25)	29 (9)	0.13[a]	32 (23)*	40 (11)*	0.22[a]	33 (21)	38 (13)	0.14[a]	12 (10)	32 (10)	0.22[a]	-
**Age**	23.09 (3.57)	23.07 (3.62)	0.01 [-0.39, 0.40]	23.30 (3.38)	22.85 (3.78)	0.13 [-0.26, 0.51]	23.20 (3.48)	23.00 (3.72)	0.06 [-0.33, 0.44]	24.71 (3.62)*	22.85 (3.60)*	0.52 [-0.01, 1.04]	-
**Full-scale IQ**	105.86 (13.37)	105.23 (16.57)	0.04 [-0.35, 0.44]	107.94 (12.71)	103.16 (16.02)	0.33 [-0.05, 0.72]	104.85 (16.03)	106.82 (12.74)	-0.14 [-0.52, 0.25]	105.44 (13.06)	103.73 (14.20)	0.12 [-0.39, 0.64]	-
**SRS-2 (Self)**	66.22 (9.81)*	61.59 (10.27)*	0.46 [0.06, 0.87]	**69.31 (8.84)*** **	**59.46 (9.03)*** **	**1.10 [0.69, 1.52]**	66.94 (8.42)**	61.58 (10.72)**	0.56 [0.16, 0.95]	67.27 (10.15)	62.22 (9.87)	0.51 [-0.02, 1.03]	-
**DAWBA Anxiety**	3 (0-4)**	2.5 (0-4)**	-0.54[b]	3 (0-4)*	3 (0-4)*	-0.47[b]	3 (1-4)*	3 (0-4)*	-0.47[b]	3.5 (1-4)*	2.5 (0-4)*	-0.49[b]	-
**DAWBA Depression**	1 (0-5)**	0 (0-5)**	-0.63[b]	**1 (0-5)*** **	**0 (0-5)*** **	**-0.93[b]**	1 (0-5)**	0 (0-4)**	-0.72[b]	1 (0-5)	0.50 (0-5)	-0.26[b]	-

b) CHIP-CE (Children/ Adolescents 6-17-years)

	**Satisfaction**			**Comfort**			**Resilience**			**Risk Avoidance**			**Achievement**		
	**>1SD below**	**Within/ above 1SD**	***d* [95% CI]**	**>1SD below**	**Within/ above 1SD**	***d* [95% CI]**	**>1SD below**	**Within/ above 1SD**	***d* [95% CI]**	**>1SD below**	**Within/ above 1SD**	***d* [95% CI]**	**>1SD below**	**Within/ above 1SD**	***d* [95% CI]**
N	126	112	-	96	142	-	70	168	-	82	154	-	125	98	-
**Sex: Males (Females)**	88 (38)	85 (27)	0.07[a]	64 (32)	109 (33)	0.11[a]	48 (22)	125 (43)	0.06[a]	62 (20)	109 (45)	-0.05[a]	93 (32)	69 (29)	-0.04[a]
**Age**	13.01 (2.92)	12.61 (3.31)	0.13 [-0.13, 0.38]	12.12 (3.08)**	13.29 (3.05)**	-0.38 [-0.64, -0.12]	13.40 (3.06)	12.58 (3.11)	0.26 [-0.01, 0.54]	12.23 (2.98)*	13.10 (3.13)*	-0.28 [-0.55, -0.01]	12.77 (3.00)	12.47 (3.21)	0.10 [-0.17, 0.36]
**Full-scale IQ**	98.13 (19.22)	97.56 (19.99)	0.03 [-0.23, 0.29]	97.43 (18.83)	98.15 (20.08)	-0.04 [-0.30, 0.23]	96.95 (18.06)	98.24 (20.18)	-0.07 [-0.35, 0.22]	97.43 (18.38)	98.40 (20.13)	-0.05 [-0.32, 0.22]	**94.41 (16.66)*** **	**106.60 (18.65)*** **	**-0.69 [-0.97, -0.42]**
**SRS-2 (Parent)**	**78.20 (8.81)*** **	**72.18 (11.80)*** **	**0.58 [0.32, 0.85]**	**78.96 (9.36)*** **	**72.93 (10.93)*** **	**0.58 [0.32, 0.85]**	77.07 (9.36)	74.69 (11.18)	0.22 [-0.06, 0.51]	76.53 (9.49)	74.69 (11.35)	0.17 [-0.10, 0.44]	**77.63 (9.47)*** **	**72.17 (11.72)*** **	**0.52 [0.25, 0.79]**
**DAWBA Anxiety**	**3 (1-5)*** **	**2 (0-5)*** **	**-0.63[b]**	**3 (1-5)*** **	**2 (0-5)*** **	**-0.77[b]**	3 (0-5)	2 (0-5)	-0.10[b]	2 (0-5)	2 (0-5)	-0.06[b]	2 (0-5)	2 (0-5)	-0.12[b]
**DAWBA Depression**	**1 (0-5)*** **	**0 (0-4)*** **	**-0.61[b]**	**1 (0-5)*** **	**0 (0-4)*** **	**-0.77[b]**	1 (0-5)	0 (0-5)	-0.28[b]	1 (0-5)**	0 (0-5)**	-0.43[b]	1 (0-5)	0 (0-4)	-0.06[b]

Note: WHOQoL-BREF=World Health Organisation Quality of Life – Brief instrument; CHIP-CE=Child Health and Illness Profile; SRS-2=Social Responsiveness Scale – Second Edition; DAWBA=Development and Wellbeing Assessment; SD=Standard deviation; *d*=Cohen's *d* effect size [95% confidence intervals]. [a]Phi effect size; [b]*r* effect size for Mann-Whitney U was converted to *d* using Rosenthal (1994). *$p<0.05$; **$p<0.01$; ***$p<0.001$ (significant after Bonferroni correction; $p=0.05/54$).

Supplementary Table 3.

Regression coefficients and model fit after including sensory processing differences and ADHD symptoms in CHIP-CE regression models for the child/ adolescent group.

		Satisfaction	Comfort	Resilience	Risk Avoidance	Achievement
		**β [95% CI]**	**β [95% CI]**	**β [95% CI]**	**β [95% CI]**	**β [95% CI]**
**Demographic**	**Age**	-0.11 [-0.29, 0.06]	0.04 [-0.11, 0.20]	-0.19 [-0.36, -0.02]*	0.20 [0.04; 0.37]**	0.01 [-0.15; 0.16]
	**IQ**	-0.07 [-0.25, 0.09]	0.12 [-0.03, 0.27]	0.03 [-0.14, 0.19]	0.02 [-0.15; 0.18]	**0.33 [0.18; 0.51]*****
	**Sex**	-0.02 [-0.20, 0.15]	0.12 [-0.02, 0.29]	-0.05 [-0.21, 0.12]	-0.21 [-0.39; -0.06]**	-0.17 [-0.34; -0.01]*
**Core traits**	**SRS-2 (Parent)**	-0.13 [-0.34, 0.08]	0.03 [-0.16, 0.21]	0.03 [-0.18, 0.23]	-0.12 [-0.32; 0.09]	-0.21 [-0.39; -0.003]*
**Associated**	**SSP**	0.10 [-0.10, 0.30]	**0.26 [0.09, 0.45]*****	0.19 [-0.02, 0.37]	0.03 [-0.17, 0.23]	0.09 [-0.10, 0.28]
	**Anxiety**	-0.23 [-0.31, -0.05]**	**-0.28 [-0.33, -0.10]*****	0.04 [-0.10, 0.16]	0.15 [-0.02; 0.24]	0.11 [-0.04; 0.20]
	**Depression**	**-0.23 [-0.38, -0.07]*****	**-0.35 [-0.47, -0.20]*****	-0.14 [-0.27, 0.03]	-0.19 [-0.32; -0.02]*	-0.15 [-0.27; 0.01]
	**ADHD**	-0.01 [-0.11, 0.10]	-0.07 [-0.14, 0.05]	0.02 [-0.09, 0.11]	**-0.29 [-0.28, -0.08]*****	-0.19 [-0.21, -0.01]*
**Model fit – Total effect (R^2_{adj})**		$F_{(8, 135)}$=5.01, p<0.001***, η_p^2=0.56 (18.3%)	$F_{(8, 135)}$=10.42, p<0.001***, η_p^2=0.62 (34.5%)	$F_{(8, 135)}$=1.50, p=0.16, η_p^2=0.52 (2.7%)	$F_{(8, 135)}$=4.78, p<0.001***, η_p^2=0.56 (17.5%)	$F_{(8, 125)}$=6.59, p<0.001***, η_p^2=0.59 (25.2%)

Note: SRS-2=Social Responsiveness Scale – Second Edition; SSP=Short Sensory Profile; ADHD=Attention deficit/ hyperactivity disorder; β=standardised regression coefficient [95% confidence intervals]; F=F-test for model significance (degrees of freedom, sample size); η_p^2=partial eta-squared effect size. *p<0.05; **p<0.01; ***p<0.006 (significant after Bonferroni correction; p=0.05/8). Sensory processing differences and ADHD symptoms were not included in WHOQoL-BREF models for the adult group since a power analysis suggested a minimum sample of N=52 was required for a model with 8 independent variables to provide power of 0.8 (α<0.05; f^2=0.35) and too few adults had data for sensory processing differences (N=41)/ ADHD symptoms (N=59) after including other variables. Nevertheless, simple correlations indicated that neither sensory processing differences (r_s=0.21, p≥0.17) nor ADHD symptoms (r_s=-0.07- -0.16 , p≥0.22) were significantly associated with WHOQoL-BREF domain scores in the adult group, though there were nominal associations with Physical Health (sensory r_s=0.32, p=0.03; ADHD r_s=-0.27, p=0.04) and Environment (sensory r_s=0.41, p=0.03).
